# Advanced Quality and Comparability Assessment of mRNA-Loaded Lipid Nanoparticles: Absolute Size Distribution Profiles and Structure from AF4-Coupled Light and X‑ray Scattering Measurements

**DOI:** 10.1021/acs.analchem.5c05911

**Published:** 2026-02-10

**Authors:** Bastian Kolb, Melissa Graewert, Roland Drexel, Florian Meier, Justin Raab, Christoph Wilhelmy, Thomas Nawroth, Dmytro Soloviov, Heinrich Haas, Peter Langguth

**Affiliations:** † Department of Biopharmaceutics and Pharmaceutical Technology, 153610 Johannes Gutenberg-University, Staudinger Weg 5, Mainz 55128, Germany; ‡ 128803 European Molecular Biology Laboratory, Hamburg Unit, Notkestrasse 85, Hamburg 22607, Germany; § Postnova Analytics GmbH, Rankingstrasse 1, Landsberg am Lech 86899, Germany; ⊥ NeoVac Ltd, West Central 127 Olympic Avenue, Milton Park OX14 4SA, United kingdom

## Abstract

The success of mRNA lipid nanoparticles (LNPs) used in the COVID-19 vaccines has demonstrated the significance of pharmaceutical products utilizing nanoparticle-based drug delivery systems in global healthcare. For the assessment of the safety, efficacy, and quality of these complex, multicomponent systems, it is important to consider not only the properties of the individual components but also their colloidal organization. There is a need for standardized methods to fulfill requirements for application in regular quality control, providing information on these properties in pharmaceutical products. To gain insight into size and size-resolved quality attributes of LNPs, we apply asymmetrical flow field-flow fractionation (AF4) coupled in-line with synchrotron small-angle X-ray scattering (SAXS) measurements, multiangle light scattering (MALS), and UV absorption measurements. We propose model-free algorithms for the analysis of light scattering and X-ray scattering data to obtain quantitative size distribution profiles from both methodologies. The approach is equally applicable for SAXS and MALS data, but SAXS additionally provides detailed, size-resolved insight into internal structure. Information on various quality-indicating parameters for the size-fractionated samples is obtained, including drug loading, internal organization, and particle shape. Since this approach does not require any model assumptions to obtain structural, quality-indicative information from experimental data, it is ideally suitable to evaluate the comparability of results from different systems and different laboratories. This makes it a valuable extension to the regular quality control panel for pharmaceutical nanoparticles, and it should be considered as a standard method in the pharmacopoeias.

## Graphical abstract

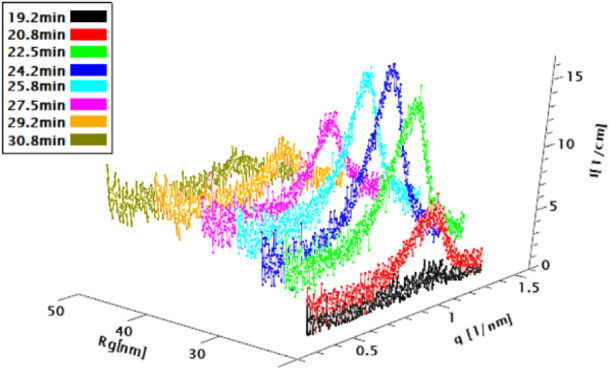

## Introduction

Despite being the focus of extensive research and development, many challenges regarding the control of manufacturing and quality aspects of LNPs, as well as other nanoparticulate drug products, remain to be solved.^1,2^ Efforts by the authorities to harmonize control strategies for mRNA-based vaccines are ongoing to make recommendations that can be accepted into widely recognized control panels, including the U.S. and European pharmacopoeias.^3^ So far, these approaches are strongly focused on the mRNA drug substance (DS) as the active pharmaceutical ingredient (API) and the chemical properties of the individual components in the final drug product (DP). However, LNPs are complex colloidal products, being assembled from a large number of molecules, driven by noncovalent (electrostatic) forces. Properties like size and structure may vary depending on conditions during manufacturing and storage, with potential influence on activity and safety. For comprehensive quality control, these coherencies need to be better understood and quality-indicative parameters need to be identified. For example, it has been reported that “empty” particles may coexist with those comprising mRNA, and the impact on quality and safety in products containing empty and loaded vesicles should be considered.^4^ Certain structural features, such as “blebs”, which have been observed at various fractions of the product, have been discussed in the context of activity.^5–7^ Also, chemical stability, such as regarding hydrolysis or adduct formation between molecular moieties, may depend on aspects of molecular organization inside the particles.^7,100^

These criteria are considered only to a very limited extent in the current control strategies. For example, size distribution profiles and size-dependent quality aspects of the particles are not part of regular quality control. The prevailing method for size determination is currently dynamic light scattering (DLS), applied as an ensemble method (e.g., cuvette measurements). Data analysis is done using a formalism^8^ which assumes that particles are mathematically monodisperse, and only limited information on the size characteristics is obtained, particularly for DP with a high polydispersity.^9,10^

A variety of methods to determine size distribution profiles are available, including analytical ultracentrifugation, flow cytometry,^10^ microscale thermophoresis (MST),^11^ Taylor dispersion analysis,^12^ nanoparticle tracking analysis (NTA), or electron microscopy techniques. All these techniques have the advantage that subsets or even individual particles are observed, which allows more accurate data analysis in comparison to cuvette DLS measurements. Analytical ultracentrifugation is a high resolution, label free method, where particles are separated by their sedimentation coefficient. It allows to deduce the size using assumptions on density and shape of the particles. Flow cytometry based techniques, like the commercially available devices for nanoflow cytometry (Nano-FCM) analyze scattering and fluorescence of individual particles in highly diluted systems. Careful calibration is required and reference standards are required to obtain accurate size information. Also, particles which are not intrinsically fluorescent need to be labeled. MST tracks nanoparticle movement on the basis of optical properties on the application of a thermal gradient from which indirectly size shape or morphology are deduced. In NTA the Brownian motion of individual particles is analyzed to obtain the diffusion coefficient and thus the hydrodynamic radius (using the Stokes–Einstein equation). It requires highly diluted samples. In principle, size distribution profiles and particle number concentrations can be obtained, but the strong dependence of the scattering intensity from the particle size makes it difficult to get reliable quantitative profiles for samples with a high polydispersity. Electron microscopy is a well-established technique which also allows to analyze size and structure of individual particles, but it requires complex sample preparation and cannot be done in a native environment. These methods often lack direct comparability and clear analytical routines for data analysis, which makes it difficult to apply them in regular quality control for pharmaceutical products.

One characterization method which has been demonstrated to be versatile for determining size distribution profiles in nanoparticulate pharmaceuticals is asymmetrical flow-field flow-fractionation (AF4). Nanoparticles are separated as a function of their hydrodynamic size, and the size-separated fractions can be analyzed by in-line coupled detectors, including multiangle light scattering (MALS), dynamic light scattering (DLS), UV, fluorescence or differential refractive index (RI) detectors. Recently, we and others have coupled AF4 to SAXS at synchrotron facilities, which allowed for the extraction of quality-indicative information on size-resolved structural characteristics.^13–15^

It has been shown that structure analysis by SAXS, along with complementary methods such as small-angle neutron scattering (SANS) and electron microscopy, can reveal valuable information on the internal structure and the detailed organization inside mRNA nanoparticles.^16^ Information on local distribution, composite patterns, core–shell organization, bleb structures, and dynamic characteristics of excipient and water molecules, which are of relevance for stability can be determined.^6,19^

When investigating mRNA lipoplex nanoparticles, such as those currently in development for cancer immunotherapy, with SAXS coupled to AF4, previously we could quantitatively determine parameters such as the fraction of free mRNA, the size distribution profile and internal structure, as well as drug loading.

Here, we have extended this approach and developed a standardized protocol for data analysis, which is equally applicable for X-ray and light scattering data. The analysis route provides product characteristics directly from experimental data by straightforward mathematical formalisms, and allows for the determination of radius of gyration, *R*_g_, mass fraction and particle number for the size-separated fractions, using either SAXS or MALS data. This information can be combined with data from other detectors that are coupled in-line. Thus, detailed, quantitative size resolved information on quality attributes is obtained.

In our proposed work process, initial characterization is performed using SAXS, which provides a reliable reference for interpreting MALS data. Once validated against SAXS, the method can be applied using MALS data alone. This makes it compatible with standard AF4-MALS laboratory setups, without the necessity of coupling to (synchrotron) SAXS measurements. As a result, researchers can adopt a more accessible and cost-effective workflow, leveraging MALS in routine lab environments while still benefiting from the structural insight initially gained through SAXS. At workflow diagram to better understand the process of data treatment can be found in the Supporting Information Figure S5.

## Experimental Section

### Materials

1,2-Dioleoyl-*sn*-glycero-3-phosphorylethanolamine (DOPE) and *N*-palmitoyl-sphingosin-1-succinyl­[methoxy­(polyethylene glycol)­2000] (C16-PEG-2000 Ceramid) were purchased from Avanti Polar Lipids (Alabaster, AL). Ionizable lipid heptatriaconta-6,9,28,31-tetraen-19-yl-4-(dimethylamino)-butanoate (DLin-MC3-DMA) was bought from MedChemExpress (Monmouth Junction, NJ). Cholesterol was ordered from Merck (Darmstadt, Germany). All lipid stock solutions were freshly prepared. Milli-Q water was prepared using a MILLI-Q Reference A^+^ system. Water was used at a resistivity of 18.2 MΩ cm and total organic carbon <5 ppm. Citric acid, ethanol, HEPES and 100 nm polystyrene size standard (stock concentration 10 wt %) were purchased from Merck (Darmstadt, Germany). The specified size by the manufacturer using TEM was 100 nm ± 7 nm.

GMP-graded eGFP-mRNA 0.5 mg/mL stock solution was purchased from eTheRNA immunotherapies NV (Niel, Belgium).

The data analysis and creation of the figures was performed with QtiPlot 1.1.8 developed by Ion Vasilief (Bucuresti, Romania). Additionally, for SAXS data analysis ATSAS 4.0 from BioSAXS (Hamburg, Germany) and for AF4 data interpretation NovaAnalysis from Postnova Analytics (Version 2408, Landsberg am Lech, Germany) were applied.

### Methods

#### LNP Formulation

The lipid nanoparticles were prepared by microfluidic mixing using the NanoAssemblr Ignite platform (Nanoassemblr, Precision Nano-Systems Inc., Vancouver, BC, Canada). Mixture A contained GMP-graded eGFP-mRNA 0.5 mg/mL stock solution diluted with 0.1 M citric acid buffer pH = 5.0 to a concentration of 0.1 mg/mL. By mixing lipid stocks solutions containing Dlin-MC3-DMA, DOPE, cholesterol, and C16-PEG-2000 and diluting the mixture to a total lipid concentration of 6.48 mg/mL mixture B was prepared. By mixing both solutions at a flow rate of 1:3 (3 and 9 mL/min) using NanoAssemblr Ignite instrument with NxGen cartridges (Precision Nanosystems, Vancouver, BC, Canada) the nanoparticles were formed.

After formulation the sample buffer was changed to 10 mM HEPES pH = 7.4 by using Amicon Ultra centrifugal filters from Merck (Darmstadt, Germany) and an Eppendorf (Hamburg, Germany) centrifuge 5804 R.

#### AF4 Measurements

AF4 fractionations were carried out on an AF2000 MT AF4 system (Postnova Analytics, Landsberg am Lech, Germany (PN)). The AF4 system included an autosampler (PN5300) and in-line UV detector (PN3211) and MALS (PN3609) detector. The instrument control and data acquisition were managed by the Becquerel software (Version 2.2.0.1) as described in Da Vela et al. 2025.^17^ The polystyrene beads (PS100) were suspended in water/detergent solution (0.0125% (v/v) NovaChem, PN) at a final concentration of 1% (w/v). 150 μL of this suspension were subjected to fractionation using AF4. For the separation, a semipreparative frit inlet AF4 channel was used with a 350 μm nominal height, 50 mm shoulder width, 5 mm hip width and 277 mm tip-to-tip length equipped with a 10 kDa regenerated cellulose (RC) membrane. The carrier liquid consisted of 0.0125% Novachem solution. The crossflow rate was initially set to 2.0 mL/min and was decreased by a crossflow decay, that was implemented by several power decays with different exponents to asymptotically reach the final constant cross-flow of 0.10 mL/min (S3). The last elution step of the fractionation method was kept constant for 10 min for the polystyrene beads measurements and 30 min for the LNP measurements, respectively. After the different elution steps a rinse step was applied with 0.5 mL/min to reduce potential memory effects. The fractionated eluent was passed through a UV detector, MALS detector (with 9 active angles), and then passed directly through the capillary for SAXS analysis in a similar manner as previously described. The MALS laser power was reduced to 20% to ensure optimal scattering intensities across the full angular range.

For the measurement of the lipid nanoparticles 220 μL of the HEPES buffered nanoparticle dispersion was injected. The fractionation was carried out with 50 mM HEPES buffer pH = 7.4 as mobile phase using the same fractionation conditions as described above. The crossflow was set to 2.0 mL/min and reached, with several power decay steps, a final crossflow of 0.1 mL/min. For the corresponding method graphs for both fractionations see S4.

#### SAXS Data Collection

AF4-SAXS data was collected at P12 BioSAXS beamline of the European Molecular Biology Laboratory (EMBL) at the PETRA III synchrotron, DESY Hamburg (Germany), using beam size of 200 × 110 μm^2^ (full width at half-maximum). The eluent of the employed fractionation technique was passed through a 1 mm quartz capillary held under vacuum. The SAXS data were recorded on a Pilatus 6 M area detector (Dectris) at a sample-to-detector distance of 6 m (LNPs) and 3 m (PS) and the wavelength λ = 0.123982 nm. Series of individual 1 s exposure X-ray data frames were measured from the continuous flow in a flow through capillary from the AF4-channel. The 2D SAXS intensities were reduced to *I*(*q*) versus *q* using the integrated analysis pipeline SASFLOW. The *q*-axis was calibrated with silver behenate, and the resulting profiles were normalized for exposure time and sample transmission. Since the experimental parameters can vary from one experiment to another the data shown in the manuscript represents single measurements (e.g., one AF4-SAXS run each). For all experiments shown reproducibility has been demonstrated in different measurements during different experimental sessions.

#### Harmonized SAXS and MALS Analysis

The AF4 technique allows separation of the intrinsically polydisperse LNPs into monodisperse fractions. The direct inline coupling of MALS and SAXS measurements from the AF4 size-separated samples provide complementary data sets on the particle structure, which, due to the well-defined (monodisperse) particle characteristics, allow for accurate data analysis. We applied harmonized formalisms for SAXS and MALS data analysis, in order to directly compare results from both types of measurements.

Both visible light and X-rays, are electromagnetic waves, described by the general formalism (eq 1):

1 $$\mathrm{E⃗}\left(x,t\right)={\mathrm{E⃗}}_{0}\cdot e^{i(-kx+\omega t)}$$

The electromagnetic wave, E⃗ , travels in space in positive *x*-direction with the amplitude |*E*| as a function of position, *x* and time, *t*, depending on the wave vector, *k* = 2π/λ and the angular frequency ω., $\frac{2\pi }{T}$ , with *T* the period in seconds.

Size, shape, and internal structure of the measured nanoparticles are determined from the angular dependence of the scattered radiation (Figure 1).

**Figure 1:**
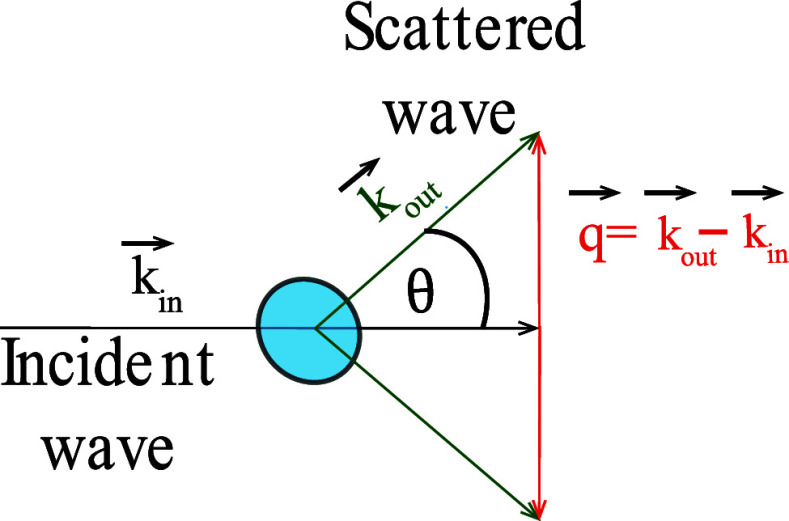
Geometry of elastic scattering.

By transcribing the scattering angles into the momentum transfer, *q* = *k⃗*_out_ – *k⃗*_in_ the angular scattering profiles for X-ray and light scattering, can be directly compared. For visible light *q* is written as (eq 2):

2 $$q=\frac{4\pi \cdot n_{0}}{\lambda }\cdot \sin\frac{\theta }{2}$$

where *n*_0_ is the refractive index of the bulk phase (water, buffer), and θ is the scattering angle. It is important to note that, historically, the definition of the scattering angle differs between small-angle X-ray scattering (SAXS) and classical light scattering techniques such as multiangle light scattering (MALS). In SAXS, the scattering angle θ is typically defined as half the angle between the incident and scattered beam, whereas in MALS, θ refers to the full angle. Thus, θ­(MALS) = 2θ­(SAXS). Here, for better comparability, we use the same formalism for MALS and SAXS.

For X-rays, the refractive index is close to one, and the expression for *q* reduces to (eq 3):

3 $$q=\frac{4\pi }{\lambda }\cdot \sin\frac{\theta }{2}$$

With λ the wavelength of the X-rays (within an order of magnitude of 0.1 nm, here 0.123982 nm) and *θ* the scattering angle.

The different wavelengths determine the *q*-values which can be assessed, which, in turn indicates the dimensions of the patterns from which information can be obtained. As a rule of thumb, the corresponding length can be estimated as $\frac{2\pi }{q}$ . The lower limit in *q* indicates the largest structures, for which information can be obtained, and the upper limit is indicative for the smallest structures. For light scattering, with the wavelength of the laser light around 500 nm (here 532 nm) and the refractive index of water, *n*_0_ = 1.33, the accessible momentum transfer, q, is limited to a maximum of 0.035 nm^–1^ (since sin θ/2 cannot be greater than one).

The accessible *q*-range for a typical SAXS measurement is determined by the detector size and distance from the sample and usually spans from about 0.01 nm^–1^ and about 10 nm^–1^ Extension to smaller values (USAXS) and larger (WAXS) values is possible, but either the *q*-range is then limited in the other direction, or additional detectors are necessary. In any case, the *q*-range covered by SAXS can extend to higher values than with MALS, therefore allowing to obtain structural information at lower distances. MALS data can be an extension to the SAXS data at the lower end of momentum transfer, where, under usual experimental settings, an overlap between the *q*-range for X-ray and light scattering can be realized, which is helpful for direct comparison of SAXS and MALS data.

As a second step of harmonization, we use the concept of the scattering length, b, which indicates the ratio between the incident and the scattered wave. It is common from neutron scattering data analysis, and for comparing X-ray and neutron scattering intensities. When an incident wave with the energy flux per area unit, *I*_0_ = |*E*_0_|^2^, interacts with the scatterer, it gives raise to a secondary electromagnetic wave, *E*_s_, with an intensity of the scattered wave, *I*_s_ = |*E*_s_|^2^, proportional to the incident wave intensity, and the squared scattering length *b* (eq 6) where *r* is the distance between detector and sample (eq 4).

4 $${|E_{s}(\theta )|}^{2}\cdot r^{2}={|E_{0}|}^{2}\cdot {(b)}^{2}\left(\frac{1+\cos^{2}\theta }{2}\right)$$

The term in the bracket reflects the spatial distribution of the polarization vector of the incident electromagnetic radiation. For both, X-rays and visible light, the scattering length, or as outlined below, the scattering length density is a parameter which is specific for the properties of the scattering particles.

For X-rays, scattering arises from interactions with electrons, where the scattering intensity of a single electron is given by the Thompson scattering length of the electron, *r*_el_, and the scattering length of one electron for X-rays, *b*, is given as (eq 5):

5 $$b_{e}=r_{\mathrm{el}}=\frac{e^{2}}{4\pi \varepsilon_{0}m_{e}c^{2}}$$

with *e* the elementary charge, *m*_e_ the electron mass, *c* the speed of light, and ε_0_ the permittivity of free space.

The relevant parameter for SAXS data analysis is the scattering length density for X-rays, ρ_e_, (eq 6), where for practical reasons mostly only the electron density is used

$$\rho_{e}=\frac{\sum b_{e}}{V}$$ 6

For light scattering, due to the lower photon energy compared to X-rays, the photons interact mostly with the outer part of the electronic cloud of an atom. The incident electromagnetic wave induces an electric dipole moment, *μ⃗*_ind_, proportional to the polarizability α, which gives rise to the scattered wave proportional to α (eq 7).

7 $${\mathrm{\mu ⃗}}_{\mathrm{ind}}∼\alpha \cdot {\mathrm{E⃗}}_{0}∼{\mathrm{E⃗}}_{s}$$

In a common notation the scattered intensity is given by the so-called Rayleigh ratio, which for the present context is written as (eq 8):

8 $${|E_{s}(2\theta )|}^{2}\cdot r^{2}={|E_{0}|}^{2}\cdot \alpha^{2}\cdot \frac{\pi^{2}}{\epsilon_{0}^{2}\lambda^{4}}\cdot \left(\frac{1+\cos^{2}\left(\theta \right)}{2}\right)$$

Therefore, in analogy to the formalim in (eq 4), the scattering length for visible light, *ρ*_l_, can be given as (eq 9):

$$(b_{l})=\alpha \cdot \frac{\pi }{\epsilon_{0}\lambda^{2}}$$ 9

which is the equivalent term to the equation for X-rays (eq 5). Because the polarizability is not readily experimentally accessible, it is convenient to express α by a parameter which can be more easily determined or calculated, such as the permittivity, ε, or the square of the refractive index, *n*, using the Clausius-Mossotti equation, which, in the form of the Lorentz–Lorenz equation correlates directly the refractive index with the polarizability by ε_r_ = *n* ^2^ (eq 10).

10 $$\frac{N\alpha }{3\epsilon_{0}}=\frac{\varepsilon_{r}-1}{\varepsilon_{r}+2}=\frac{n^{2}-1}{n^{2}+2}$$

Here we are interested in scattering from particles in solution, where the difference in scattering length density between particle and bulk phase is relevant. For X-rays, *Δρ*_*x*_ is the difference between electron density of the particle and the bulk phase, multiplied by the Thompson scattering length. For visible light, the difference in polarizability is relevant, which is taken into account in eq 11 as the ratio between the refractive index of the particle, *n*_p_, and the bulk phase *n*_0_, $m=\frac{n_{p}}{n_{0}}$ , which results in the relative refractive index and Δ*ρ*_l_ becomes (eq 11):

$$\left(\rho_{l}\right)=(\frac{m^{2}-1}{m^{2}+2})\cdot \frac{2\pi \times n_{0}}{\lambda^{2}}$$ 11

In practical analysis of light scattering data from polymer solutions, one approximates the difference in polarizability from the refractive index increment, d*n*/d*c*, which can be easily determined experimentally (eq 12).

12 $$\delta \alpha =2\varepsilon_{0}n_{0}\left(\frac{dn}{dc}\right)\frac{V_{p}}{\nu_{p}}$$

With *V*_p_ the particle volume and ν_p_ the particle density in g/m^3^

In a general form, the scattering form particles in solution can be written as (eq 13):

$$\frac{I\left(q\right)}{I\left(o\right)}\cdot r^{2}=\frac{d\sigma \left(q\right)}{d\Omega }=N_{p}\cdot {\left(\Delta \rho \right)}^{2}\cdot V_{p}^{2}\cdot F\left(q\right)\cdot S\left(q\right)$$ 13

With dσ/dΩ the differential cross section, *N*_p_ the particle number density, Δρ the contrast, *V*_p_ the particle volume, *F*(*q*) the form factor of the particle, and *S*(*q*) the interparticle structure factor. As we consider here highly diluted samples, subsequently, we will neglect the structure factor.

A value of interest for scattering from a particle, which can be calculated for any shape and internal distribution is the radius of gyration, *R*_g_, which correlates the distances and contrasts of all scattering units (eq 14):

$$R_{g}^{2}=\frac{\int b\left(r_{i}\right)\cdot r_{i}^{2}dV_{i}}{\int b\left(r_{i}\right)dV_{i}}$$ 14

The form factor of any particle can be expanded in a power series, where the intensity as a function of *R*_g_ and *q* is given by (eq 15):

15 $$F\left(q\right)=1-\frac{2}{3!}R_{g}^{2}\cdot q^{2}+\frac{2}{5!}R_{g}^{4}\cdot q^{4}-...$$

At very low *q* (*q*·*R*_g_ < 1) the power series can be truncated after the first term (Debye approximation), which contains the factor *R*_g_^2^ *q* ^2^.

This leads, in the simplest case, to a correlation comprising the intensity, together with the particle number *N*_p_, volume *V*_p_, contrast and radius of gyration of the scattering particle (eq 16):

16 $$I\left(q\right)∼N_{p}\cdot V_{p}^{2}\cdot {\Delta \rho }^{2}\cdot \left(1-\frac{1}{3}{\left({q\cdot R}_{g}\right)}^{2}\right)$$

With further approximation (eq 17):

17 $$1-\frac{1}{3}{\left({q\cdot R}_{g}\right)}^{2}\approx e^{-1/3\cdot {\left({q\cdot R}_{g}\right)}^{2}}$$

One obtains *R*_g_ from a plot of ln­(*I*(*q*)) vs *q* ^2^ (Guinier approximation, eq 18):

18 $$\ln\left(\frac{I\left(0\right)}{I\left(q\right)}\right)∼-\frac{R_{g}^{2}}{3}{\cdot q}^{2};,q\cdot R_{g}<1$$

which is valid for *q* · *R*_g_ < 1 and gives the radius of gyration from the slope of a plot of ln­(*q*) vs *q* ^2^. It may be noted that also any internal (smaller) structure with defined size leads to a similar correlation, but at higher *q*-range.

Extrapolating the intensity to *q* = 0, (*I*(0)) gives information on scattering moieties, with *N*_p_ number of particles per cm^3^, *V*_p_ the volume of particles in cm^3^, and Δρ the scattering length contrast between bulk phase and particle (eq 19).

$$I\left(0\right)=N_{p}\cdot {V_{p}^{2}\cdot (\Delta \rho )}^{2}$$ 19

Data sets from X-ray and light scattering from the size-resolved samples can be directly compared. The *I*(0) values are different, but by dividing by the contrast (Δρ)^2^, very similar numbers should be obtained. Differences still can arise, for example, from nonisotropic distribution and different contrast profiles of certain molecular moieties. For example, in a core–shell organization, a component can have a higher electron density compared to the polarizability, or vice versa. This can result in different size profiles and, consequently differences in *I*(0). As the particle radius enters to the power of six (*V*_p_)^2^ into the formalism small size changes have a very strong influence on *I*(0). It opens, on the other hand, options to gain further insight into particle characteristics. For example, if the ratio between *I*(0) for X-rays and light changes over the elution time, this points toward changes in molecular organization or composition. All constants used for the calculations in this manuscript are given in Table 1:

**Table 1:** Constant Parameters Used for Calculations

Parameter	Polystyrene beads	mRNA-LNP
Refractive index *n*0 (bulk)	1.333	
Refractive index *n* (particle)	1.59	-
Relative refractive index m	1.19	-
Wavelength λ(MALS) [m]	5.32 × 10^–7^	
Particle density (8% mRNA) ν [g/m^3^]	1.05 × 10^6^	1.05 × 10^6^
Lipid density ν[g/m^3^]	-	1.00 × 10^6^
mRNA density ν [g/m^3^]	-	1.60 × 10^6^
Avogadro number *N*_A_ [1/mol]	6.02 × 10^23^	
Refractive index increment d*n*/d*c* [m^3^/g]	2.45 × 10^–7^	1.3304 × 10^–7^
Electrons of the monomer *z* [e.u.]	56	-
Molecular weight of the monomer M [g/mol]	104.15	-
Electron density particle *p* [e.u./m^3^]	3.40 × 10^29^	3.86 × 10^29^
Electron density water *p*_0_ [e.u./m^3^]	3.35 × 10^29^	
Difference electron density Δ*p* [e.u./m^3^]	5.43 × 10^27^	5.14 × 10^28^
Optical constant *K*′ (MALS)[1/m^4^]	8.76 × 10^26^	
Thompson scattering length Tl [m^2^]	6.65 × 10^–29^	
Radius of an electron e [m]	2.82 × 10^–15^	

#### Extended SAXS Analysis

X-ray scattering provides information at a much larger *q*-range as outlined above. Analysis of the full SAXS curves allows to obtain further information on particle characteristics, including the internal particle structure at smaller length scales.

One option is to calculate the scattering curve for a given model for the particle shape over a wider *q* range. For the simplest special case of a solid sphere, the form factor can be written as (eq 20):

20 $$F_{\mathrm{sphere}}\left(q\right)=\lfloor 3\cdot \frac{\sin\left(qR\right)-qR\times \cos\left(qR\right)}{{\left(qR\right)}^{3}}\rfloor^{2}$$

In that case, the scattering curve is characterized by a sequence of minima, where the first minimum is at about *q× R* = 4.66. Therefore, in addition to Guinier analysis, the first minimum position directly allows determination of the particle size in an independent manner, with higher accuracy, as more data points can be considered, provided scattering data up to the respective *q*-value are available.

The volume of the particles, *V*_p_ can be obtained in an independent approach from the X-ray scattering curves using the formalism (eq 21):

21 $$V_{p}=\frac{2\cdot \pi^{2}\cdot I\left(0\right)}{\int_{0}^{\infty }q^{2}I\left(q\right)dq}$$

In addition to particle size and shape, also important information on the internal structure is obtained. When patterns with a defined repeat order are present, this leads to Bragg peaks. Here we apply Lorentzian profiles to determine peak position, peak width and peak area. From the Bragg peak position, *q*_c_, the repeat distance of the scattering moiety, *d,* can be calculated using the Bragg equation (eq 22):

22 $$d=\frac{2\pi }{q_{c}}$$

From the peak width, *w*, the correlation length, ξ, inside the ordered stacks can be calculated. Assuming liquid crystalline organization, ξ can be defined as the distance, at which the positional correlation decays to the value 1/*e* and is given as (eq 23):

23 $$\xi =\frac{2}{w}$$

The area of the peak is proportional to the total amount of material present in the respective state of organization. One can therefore compare the total amount of the particles (total particle volume), and the amount of the ordered material inside the particles as a function of size and check if the relative particle composition changes with size.

Further to that, one can also analyze the decay of the scattering intensity as a function of *q*, which provides information on the packing constraints of the particles, which allows to get information on the compactness, or, more generally, the fractal dimension of the particles.

We analyzed the intensity decay in the region between the q-range applied for the Guinier plots and the Bragg peak position using a power law, where *x* is denoted as the fractal dimension (eq 24):

$$I\left(q\right)∼I_{0}\cdot q^{-x}$$ 24

For particles with smooth surfaces, an exponent between 3 and 4 is observed, consistent with the Porod law. While slopes with lower decay, in the range of 2–3, are indicative for particles with a rougher surface, reflecting a higher fractal dimension.^23^

## Results and Discussion

### Data Analysis Approach for Obtaining Quantitative Size Distribution Profiles

For our study, particles were separated by the AF4 technique prior to the MALS/SAXS measurements, which ensures that fractions entering the various detector systems are sufficiently monodisperse. All detectors, including MALS and SAXS, were directly in-line coupled to the eluting size-separated sample.

Because identical monodisperse samples were investigated with MALS and SAXS, we have aligned the data analysis protocols for both methods to allow for best comparability between the results.

As a first step, we express the angular dependence of the scattering intensity as a function of the momentum transfer, *q*, which makes the profiles independent from the used wavelength (see eq 2 and eq 3).

Due to the difference in wavelengths (here 0.124 nm for X-rays and 532 nm for MALS), light scattering extends to lower *q* values, but with a limited dynamic range, as with the physical maximum value of Θ = 180° *q* is still low. Although SAXS is usually measured up to a few degrees only (here up to about 5°), it covers a higher *q* range, therefore providing higher structural resolution (information smaller distances) than MALS.

There is the option to adjust the *q*-range toward the lower and higher end (ultrasmall-angle X-ray scattering, USAXS, wide-angle X-ray scattering WAXS) and provide much higher structural resolution (e.g., providing structural information down to smaller distances) than MALS.

The lower *q* range covered by MALS could in principle be detected also by USAXS, but it would require a much larger detector distance and limit accessibility for the higher end. Therefore, both methods, MALS and SAXS, complement each other well with their provided structural information. Importantly, a certain overlap of the covered *q*-range can be realized, which is helpful for direct comparability between both measurements.

As a second step, we calculate the absolute scattering intensities for both methods by applying required calibrations (see Methods and Materials).

Third, we express the scattered intensity of the samples for both wavelengths by the same formalism, using the concept of scattering length, *b*, or scattering length density *b*/*V* = ρ, which gives the relation between the intensity of the scattered and the incident wave for a given type of radiation (see Methods and Materials). It is usually applied in neutron scattering analysis, and for comparison with X-ray scattering. For X-rays, the scattering intensity scales with the electron density, while for light scattering the polarizability, with the refractive index as a coupled and experimentally accessible value, is relevant. The numerical values depend on the composition of the samples which we measure or estimate for the expected particle composition, for better quantitative comparison of the data sets.

Having brought MALS and SAXS data into the same format, we use the Guinier approximation^18^ for determination of the radius of gyration, *R*_g_, and the extrapolated scattering intensity at zero scattering angle, (*I*(0), of the samples from both data sets (eq 18) from (eq 25):

25 $$\ln\left(I\left(q\right)\right)=\ln\left(I\left(0\right)\right)-q^{2}\times \frac{R_{g}^{2}}{3}$$

*I*(0) contains information on the particle volume *V*_*p*_, particle number concentration *N*_p_, and the scattering length density contrast, Δ*Ρ* of the particles in the medium (eq 19, see Methods and Materials for more details).

Assuming a certain particle shape (i.e., in the simplest case a solid sphere) the volume per particle *V*_p_, can be calculated from *R*_g_. The contrasts, Δρ, can be reasonably well estimated from the molecular properties of the particles’ constituent molecules or determined by independent measurements, for example by measuring the refractive index gradient in case of MALS (see Methods and Materials).

From this, the particle number per volume unit, *N*_p_, is obtained, and, together with the particle density (measured independently or estimated), the mass concentration (e.g., in μg/mL) of the particles for the size faction at the determined *R*_g_. The contrasts can be reasonably well estimated from the molecular properties of the particles’ constituent molecules or determined by independent measurements.

Having applied correct parameters for both methods, directly comparable, in the best case equivalent results should be obtained. Differences between the results from SAXS and MALS may be due to limitations of the accuracy by which the scattering length density can be determined, or they may point toward differences in inhomogeneous contrast distributions for the particles, such as effects of a core–shell organization or overall anisometry of the particle. Note that small differences in the particle size (e.g., when the relative contributions of electron density and refractive index are different for a shell structure) lead to high relative deviations, as the radius enters with its sixth power into *I*(0). Further data analysis, referring to different particle shape, conformation and internal structure is possible, and will be outlined in the following sections.

### Proof of Concept with Polystyrene Size Standard

To validate this analytical approach, we conducted a proof-of-concept experiment by analyzing a polystyrene size standard, with a certified size of 100 nm in diameter determined by transmission electron microscopy (TEM). As expected for a monodisperse standard, the AF4 fractogram, results in a single peak (Figure 2A) for both the X-ray (red) and the light scattering (black).

**Figure 2:**
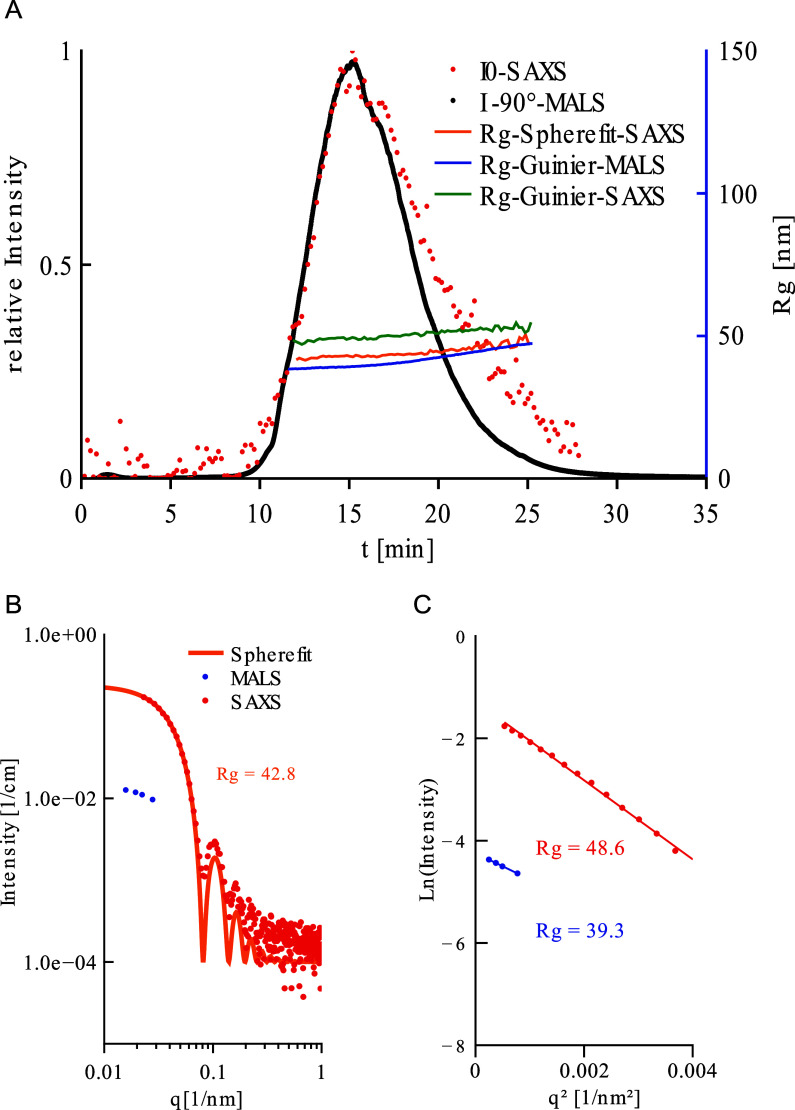
AF4-SAXS/MALS Analysis of 100 nm Polystyrene (PS100) Beads.150 μL of the respective suspension were fractionated using AF4 (for further conditions see Methods and Materials part). (A) AF4 fractogram showing 90°light scattering (black) and *I*(0) from SAXS Guinier analysis (red). Calculated radii of gyration as a function of elution time are shown in orange, blue, and green. (B) Scattering data for MALS (blue dots) and SAXS (red dots) at the peak maximum (15.1 min elution time) plotted together as a function of the momentum transfer *q*. The orange line gives the result of a solid sphere fit, resulting in the particle size of 110 nm (*R*_g_ = 42.8 nm). (C) Guinier plots of the SAXS (red data points) and MALS (blue data points) at peak maximum. Linear fits and resulting *R*_g_ values as indicated. Several SAXS curves at different time points over the of elution period are given in the Supporting Information Figure S1.

Figure 2B displays the (background adjusted) scattering data of both MALS and SAXS as a function of *q* (collected for the time point at the peak maximum (*t* = 15.1 min)). MALS data (blue data points) cover a narrow *q*-range at the lower end of the SAXS data (red dots), which extend to much higher *q*-values, having an overlapping section with MALS. The difference in intensity results from the above-described differences in contrast, namely electron density and polarizability, for the two wavelengths. The shape of the SAXS curve is in accordance with the form factor of a monodisperse solid sphere, showing several clearly pronounced fringes. The orange line shows the fit of the solid sphere model, with the fitted diameter of about 110 nm, which is well in accordance with the specified particle diameter of 100 nm ± 7 nm. For better comparability with the Guinier fits, in Figure 2B the corresponding radius of gyration, *R*_g_ = 42.8 nm is given. The results of the Bragg peak and Guinier analyses are summarized in Table 2:

**Table 2:** Parameters Derived from Guinier Analysis of Polymer Standard and LNPs

Parameter	Polystyrene beads	mRNA-LNP
		
Intensity at 0° angle at peak of the fractogram *I*0(SAXS) [1/m]	2.80 × 10^1^	3.55 × 10^1^
Radius of gyration *at peak of the fractogram R*_g_(SAXS)[m]	4.86 × 10^–8^	2.75 × 10^–8^
Scattering length density *ρ*(SAXS) [1/m^4^]	2.39 × 10^27^	1.76 × 10^29^
Intensity at 0° angle at peak of the fractogram *I*(0)(SAXS-Sphere) [1/m]	2.40 × 10^1^	1.74 × 10^1^
Radius of gyration at peak of the fractogram *R*_g_(SAXS-Sphere)[m]	4.28 × 10^–8^	3.40 × 10^‑8^
Particle number concentration (pnc) *at peak of the fractogramN*_p_ (SAXS) [1/m^3^]	1.09 × 10^16^	2.18 × 10^17^
Partricle number concentration with *R*_g_ from Spherefit at peak of the fractogram(SAXS)[1/m^3^]	2.35 × 10^16^	7.21× 10^18^
Intensity at 0° angle *at peak of the fractogram I*0(MALS)[1/m]	1.44	2.35 × 10^–1^
Radius of gyration *at peak of the fractogram R*_g_(MALS)[m]	3.93 × 10^–8^	3.31 × 10^–8^
Scattering length density *ρ*(MALS)[1/m^4^]	5.78 × 10^25^	1.70 × 10^25^
Particle number concentration *at peak of the fractogram N*_p_(MALS) [1/m^3^]	3.42 × 10^16^	1.29 × 10^17^

In Figure 2C, Guinier plots (eq 18), which show the natural logarithm of the scattering intensity as a function of the square of the momentum transfer, *q*, are given for the SAXS (red) and MALS (blue) data at the same point of time. In both cases the linear correlation is consistent with the presence of monodisperse particles of defined sizes. The values for *R*_g_ are in accordance with the expectations, with slight differences between the data sets (for fit parameters and the according confidence levels see Supporting Information Table S3). The result from MALS, with an *R*_g_ of 39.3 nm, corresponds to the diameter of a solid sphere of 101 nm diameter, perfectly in accordance with the specified value by the manufacturer. The SAXS result is slightly higher, with *R*_g_ = 48.6 nm, corresponding to a diameter of 125.5 nm for a solid sphere. Notably it is also larger than the result from fitting the solid sphere to the full SAXS data over the full q-range, where 110 nm were determined.

We attribute this discrepancy in size to differences in technical conditions and contrast for the respective data. Due to the tendency of latex beads to be adsorbed to the membrane in the AF4-channel as well as to the capillary wall we had to use a mobile phase with a surfactant (NovaChem, see Materials and Methods). The presence of a detergent layer at the particle interface from the use of surfactant, which leads to a stronger effect on the electron density profile than the refractive index increment, can be the reason for larger values from the SAXS data compared to MALS. The higher deviations in the Guinier analysis can also be caused by deposits formed at the capillary wall due to radiation damage induced by the X-ray beam likely leading to aggregation or structural deterioration of the sample.^19^ These contribute particularly to scattering intensity at very low q-values. Therefore, in Guinier analysis such contamination leads to higher slope of the fitted line. For the sphere fit the whole curve is used and therefore these effects contribute less.

The green, orange and blue line in Figure 2A show the result of size determination with the three approaches as a function of elution time for the whole peak. With the slight systematic differences, the results are in very good accordance with each other, showing that all particle fractions are in a very narrow range as expected for the standard.

*R*_g_ values derived from Berry fits (which is frequently used in MALS data analysis for LNPs)^9,20^ are shown in Supporting Information Figure S1b, are consistent with the results from the different approaches to determine *R*_g_ from the scattering data are plotted together.

We used the Guinier plots also to extrapolate to the scattering intensity at zero scattering angle, *I*(0), obtaining the profiles of *I*(0) as a function of elution time (Figure 3A). The MALS (black) and SAXS (red) profiles are well in accordance with each other, where we attribute a slight tailing of the SAXS elution profile data toward higher elution time as a band broadening effect due to the relative broad capillary (1 mm). The evolution of *I*(0) taken from the solid sphere fits (orange), correlate with the MALS *I*(0) curve as well.

**Figure 3:**
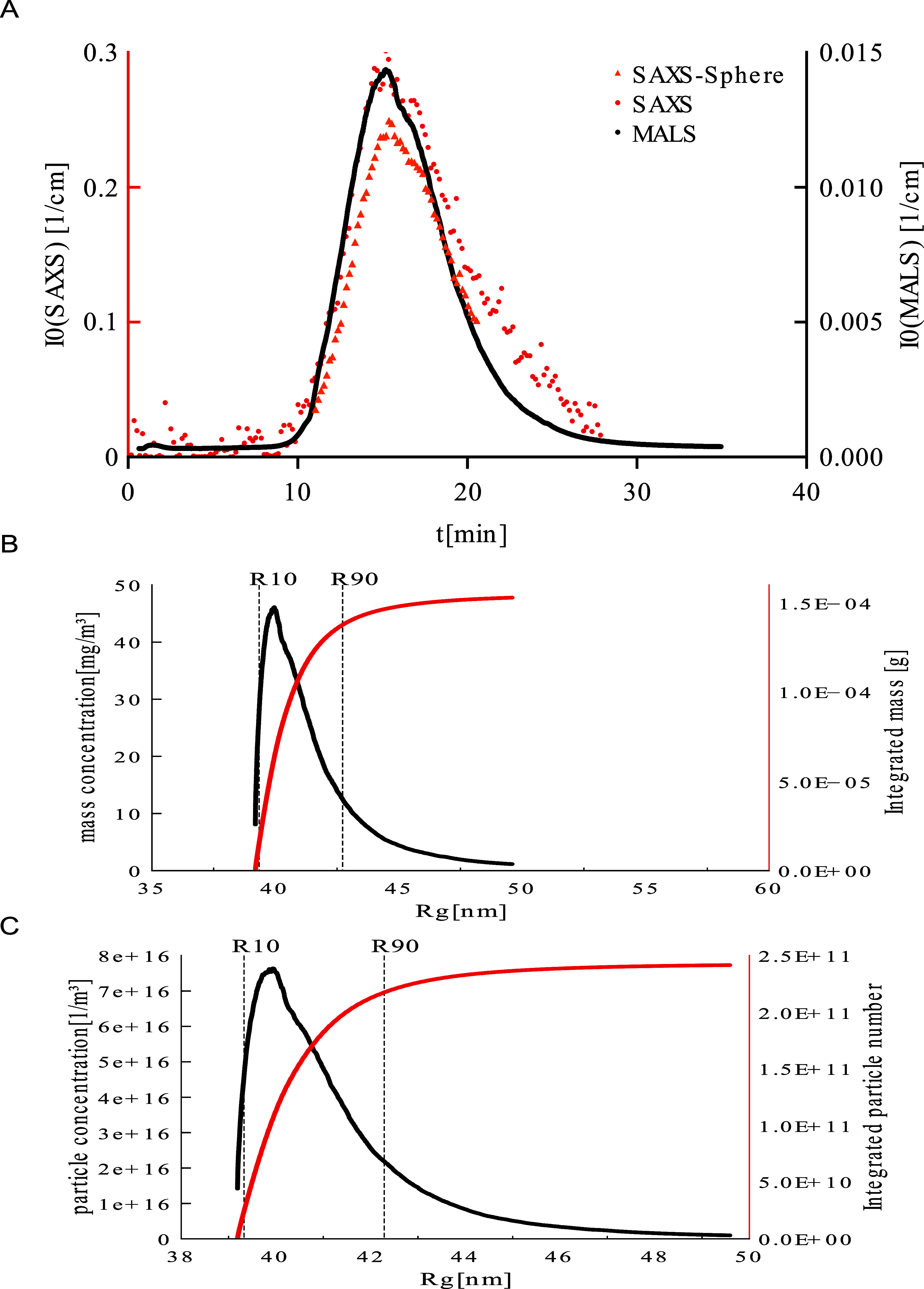
Quantification of polystyrene 100 nm particles. (A) fractogram overlaying the intensities obtained from MALS and SAXS data over the separation time. (B,C) particle concentration and mass concentration, respectively, as a function of radius of gyration with the respective cumulative curves.

As stated above, from *I*(0) and *R*_g_, information on the particle number per volume unit can be derived if the scattering length density contrast is known or estimated. Also, the absolute mass concentration is given with the estimated or known density, ν_p_, of the particles (in mass per volume unit) (see Methods and Materials). It should be noted that potential errors which may be introduced by these parameters are relatively small, as they vary only in a small range.

For calculation of the particle number and mass concentration, we analyze the MALS signal using the refractive index for polystyrene, *n*_p_ = 1.59 and water, *n*_0_ = 1.333, which can be transformed into the refractive index increment $\frac{dn}{dc}$ (see Methods and Materials) and the contrast Δ*ρ*_l_.^21^ With the particle volume, obtained from *R*_g_ assuming the solid sphere model, the evolution of the particle concentration and particle number is obtained. Figure 3B and C shows the particle number concentration [1/m^3^] and mass concentration [mg/m^3^], as differential and cumulative distributions as a function of size, here given as *R*_g_ obtained from the Guinier analysis of the MALS-data.

By integrating the curves, one obtains the cumulative distribution profiles (mass and particle number, respectively) as a function of size. With the experimental parameters and model assumptions as given above, the cumulated curves result in a total mass of 0.15 mg, in accordance with the injected mass (0.15 mg). The full recovery of the injected material demonstrates the excellent technical setup of the experiment and confirms the suitability of this approach for data analysis to determine the particle characteristics. Based on the recovered material, one can calculate quality related parameters in polydisperse systems like the quantitative fractions within certain size windows, denoted R10, R50, and R90 values. Obviously, since a size standard was used, a particularly good monodispersity was found, with a narrow size distribution between the lower and the upper limit, with R10 = 50.8 nm, R50 = 51.9 nm and R90 = 55.1 nm. The amount of material below R10 and up to R90 can be taken from the graphs with *m*(R10) = 15.23 ug, *n*(R10) = 2.32 × 10^10^ particles and *m*(R90) = 137.7 ug, *n*(R90) = 2.17 × 10^11^ particles. Therefore one can quantify the number of particles as well as the overall mass between 50.8 and 55.1 nm with *m*(50.8 nm–55.1 nm) = 122.47 ug, *n*(50.8 nm–55.1 nm) = 1.94 × 10^11^ particles. Such information is of high relevance for the assessment of nanoparticulate and microparticulate systems. So far, such information could be determined only for products with much larger particle numbers, where other analytical assays, such as counting, cryo-electron microscopy or laser diffraction are applicable. Overall, from the data obtained from the defined, monodisperse standard the validity of the procedures for data analysis can be concluded.

### Analysis of mRNA-Loaded LNPs

Having confirmed the validity of the protocols from the measurements of the polymer standards, we investigated a mRNA-LNP formulation. LNPs were manufactured with internal microfluidic protocols, using the cationic lipid MC3 and eGFP-mRNA as a cargo (see Methods and Materials). 220 μL of LNPs at a mRNA concentration of 0.1 mg/mL were injected without sample preparation to the AF4, and the resulting fractograms with signals from the different detectors are shown in Figure 4A. While light scattering signal (black line) shows a single, relatively compact peak in the elution profile, with only a small tailing toward higher elution times, a clearly pronounced shoulder toward higher elution times can be detected in the UV signal (purple line).

**Figure 4:**
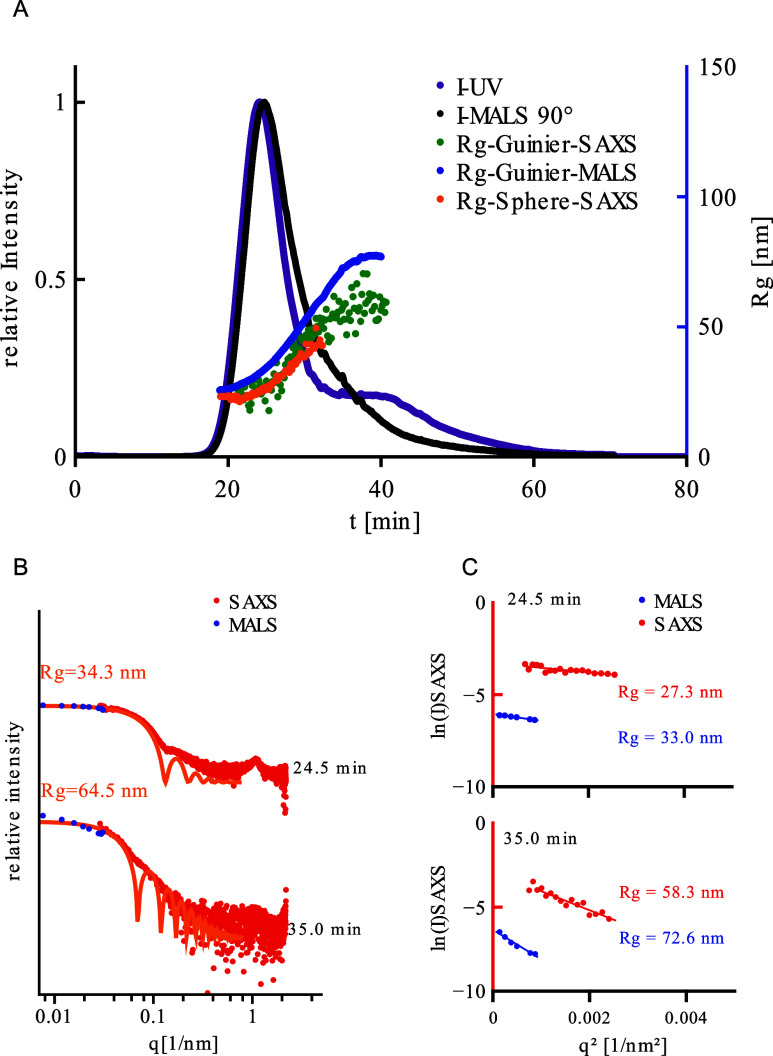
Analysis of eGFP-loaded LNP’s (N/*P* = 5). (A) fractogram, with 90° light scattering intensity (λ = 532 nm), UV (λ = 280 nm) and radius of gyration as a function of elution time. (B) conjoined display of SAXS-scattering curve and the *q* converted MALS-angles at 24.5 min (peak) and 35.0 min. Both scattering curves are displayed relatively to one another. (C) Guinier-fits of both methods.

Figure 4B shows the scattering intensity as a function of momentum transfer for MALS and SAXS together at the peak maximum (24.5 min), and in the range of the shoulder, at the elution time 35.0 min. The intensities of MALS and SAXS are normalized by their respective *I*(0) values to allow direct comparison of the two data sets. Fringes in the X-ray scattering curve (best visible for the data at earlier elution time, in the peak maximum) are discernible, indicative for compact globular particles. Fitting a solid sphere model curve (orange line) yields *R*_g_ values of 34 nm (peak) and 64 nm (shoulder).

The data displayed in blue (*R*_g_-Guinier MALS), green (*R*_g_-Guinier-SAXS) and orange (*R*_g_-Sphere-SAXS) in Figure 4A present the results from the size measurements with the respective techniques. All curves share the same characteristics, with a clear increase of the particle size as a function of elution time, in accordance with the intrinsically polydisperse nature of the nanoparticles. Results from Guinier and sphere fit from SAXS are in close accordance with each other, while the numbers from MALS are slightly higher. The offset between results from the different analytical methods are considered to be due to differences in contrast, as stated above.

One further feature which can be discerned in the X-ray scattering curves is the broad Bragg peak, with a maximum position at around 1 nm^–1^, clearly visible in Figure 4B, 24 min. It derives from internal stacks of repeating lipid and mRNA layers, which exhibit a relatively low degree of order.^22^ In Figure 5B, showing the Bragg peak in linear scale at different elution times, it can be seen that the peak position and shape remain very similar over the elution time, but the area changes substantially.

**Figure 5:**
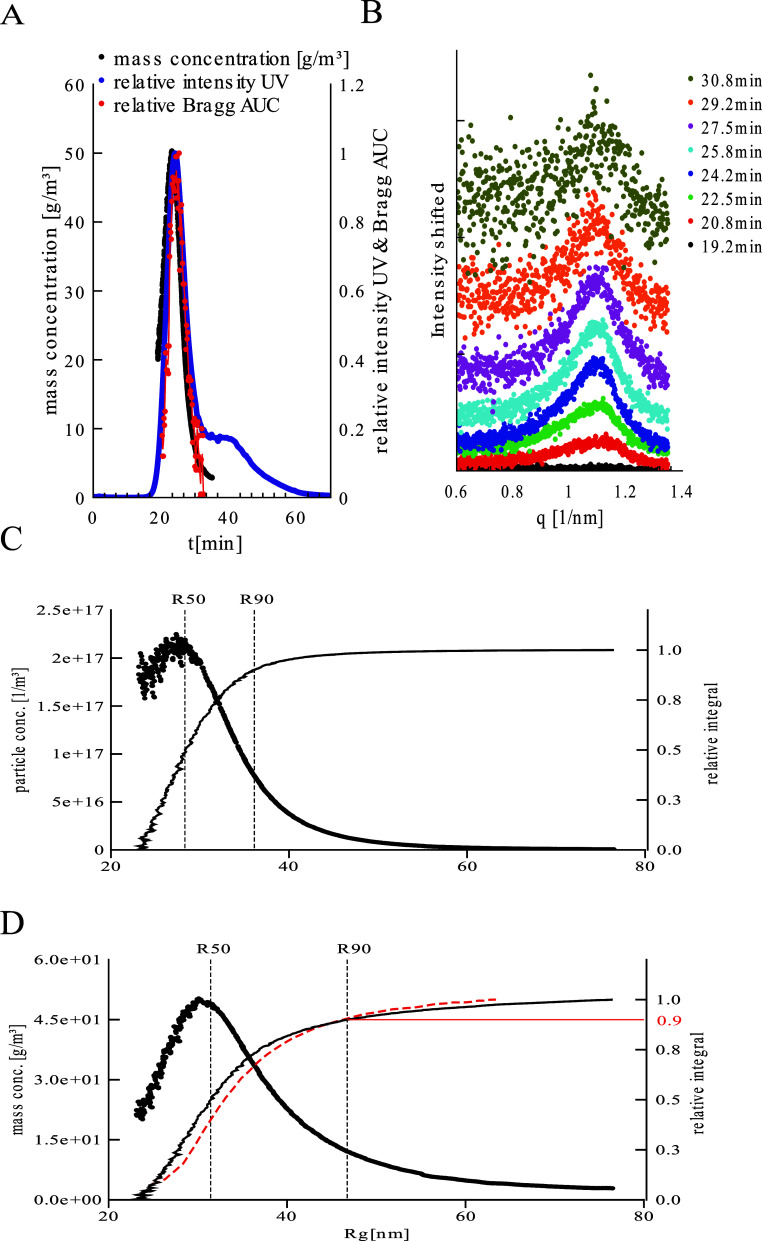
Quantification of LNPs. (A) Relative Bragg peak area (AUC) together with the mass concentration of the particles and UV signal as a function of the elution time. (B) Scattering curves in the Bragg peak region at various time points in linear scale (vertically shifted for clarity). (C and D) particle concentration and mass concentration vs particle size with the according cumulative integrals, respectively. The red dotted integrals represent the area from the Bragg peak.

In the Supporting Information Figure S2, SAXS curves over the whole elution period are given together with results from fitting Lorentzian profiles to obtain peak position, width and area. The Bragg peak information correlates with the repeat distance, the correlation length and the relative amount of material having this type of order (for details see Methods and Supporting Information Table S2). Data are in accordance with earlier findings indicative for stacks consisting of a low number of repeating lipid and mRNA layers.

As demonstrated with the polystyrene size standard, quantitative size distribution profiles can be obtained from the *I*(0) and *R*_g_ values determined by the Guinier analysis. Here, in addition, the relative amount of ordered material, derived from the Bragg peak area, can be observed in comparison to the total amount of particulate material.

Figure 5A shows the Bragg peak area together with the mass concentration of the particles as a function of the elution time. Also, the UV signal (at 280 nm) is shown, which is the sum of absorption from mRNA and cholesterol as well as scattering contributions. The profile for the total amount of particulate mass and the ordered material are similar in shape, except for the smallest particles at around 20 min, which contain a lower relative fraction of ordered material. Apparently, in very small particles, less, or no, ordered material is present. However, the shape of the UV trace suggests that a fraction of mRNA is in fact incorporated in these particles. On the other hand, at larger elution times, the total mass and the amount of ordered material show a very similar profile, and they do not display a shoulder comparable to the UV trace (around 40 min). Therefore, it can be concluded that the UV signal mainly results from scattering but does indicate the presence of substantial amounts of mRNA and lipid in that range (the scattering intensity scales with the sixth order of size, therefore very small amounts of large particles can account for a substantial contribution to the UV signal). This demonstrates that the quantitative analysis of signals from the different detectors can be valuable for deciding if certain findings in the elution profile are relevant for product quality or not.

In Figure 5C (mass concentration) and 5D, (particle number concentration) the size distribution profiles are shown, calculated by the protocols as outlined above for the analysis of the polystyrene size standard. Here, as expected, broader distribution profiles compared to the polymer standards are obtained, due to the intrinsic polydispersity of the LNPs. Quantitative values for the of the product are obtained. While the median for the particle number is at 28.3 nm, it is at 31.4 nm for the mass concentration. Similar shapes of the cumulative mass distribution profile and the profile for the ordered material (Figure 5D) indicate that the different size fractions of the particles have a similar internal structure. Both, mass concentration and Bragg peak area are very low in the range where the UV shoulder is present, therefore, the shoulder in the UV trace can be considered to result from only very small fractions of (strongly scattering) larger particles, but not a substantial second particle population with aberrating quality. This example highlights the potency of the methodology for evaluation of product quality in case of unclear results from other methods. Such information can greatly facilitate decision making on rejection or acceptance of a product when observations from other assays are unclear. Importantly, the approach can be applied in standard laboratories, including QC environment, using MALS (and other laboratory detection systems) alone. SAXS can provide valuable Supporting Information, such as on internal structure or molecular organization, or as a reference to the MALS data.

## Conclusion

It is unquestionable that better size-resolved information is required for reliable quality control of LNPs. The limited toolset of current methods, basically consisting of cuvette DLS measurements, do not give full information on size-related product quality aspects. There is a risk that undesired changes are not noticed, and when changes are observed, it can be difficult to decide to which extent they are relevant for product quality.

The high size resolution capability of AF4 enables the characterization of the complete mRNA-LNP size distribution profile. Direct insight into physical polydispersity is given and particle fractions with different properties can be identified and quantified. In the present case, a suspected larger fraction was identified, which would not have been detected by cuvette DLS (Supporting Information Figure S4).

Further to the repeat distance as derived from the Bragg peak position, as well other features of the SAXS curve, that have demonstrated to be relevant for biological activity, can be analyzed, such as surface fractality derived from the Porod exponent.

Taken together, in this manuscript we outline an approach to obtain size-resolved, quantitative, information on structure and other quality indicating attributes of pharmaceutical nanoparticle formulations. The proposed workflow comprising straightforward mathematical formalisms for analysis of MALS and/or SAXS data from size separated samples by AF4 allow direct comparison between results from different laboratories and systems, and they are suitable for being applied in regular quality control. Both instrumentation and approach for data analysis are well-suitable for being setup in a controlled environment. The information obtained reaches beyond the scope of the current standard control panel and it is highly relevant for evaluation of the quality of polydisperse pharmaceutical nanoparticle products, such as LNPs. Different fractions in the product can be identified, quantified, and evaluated regarding their relevance for the product quality. Hence, this methodology opens a promising avenue for a direct comparison between results from different laboratories and systems, rendering this approach ideal for regular applications in quality control. It should therefore be considered in the respective pharmacopoeias as part of the standard assays for better control of product quality.

## Supplementary Material



## Notes

Figure S1 and Figure S2: Results of relevant fits of MALS and SAXS data; Figure S3: AF4 separation method; Figure S4: Results from batch DLS-measurement; Figure S5: Workflow diagram; Table S1: Scattering angles to scattering vector conversion; Table S2: Parameters from Bragg peak analysis; Table S3: Fit parameters from Guinier plots with confidence levels (PDF)
