# Supplementary material for: Advanced Quality and Comparability Assessment of mRNA-Loaded Lipid Nanoparticles: Absolute Size Distribution Profiles and Structure from AF4-Coupled Light and X‑ray Scattering Measurements
Source: Anal Chem. 2026 Feb 10;98(7):5271–83. doi: 10.1021/acs.analchem.5c05911 (PMC12937060; doi:10.1021/acs.analchem.5c05911)
Supplement: Supplementary file 1 [file ac5c05911_si_001.pdf]

Supporting information

Advanced quality and comparability assessment of mRNA loaded lipid nanoparticles:

Absolute size distribution profiles and structure from AF4-coupled light and X-ray scattering measurements

Bastian Kolb[a], Melissa Graewert[b], Roland Drexel[c], Florian Meier[c], Justin Raab[a], Christoph Wilhelmy[a], Thomas Nawroth[a], Dmytro Soloviov[b], Heinrich Haas[a] and Peter Langguth[a*].

[a]Department of Biopharmaceutics and Pharmaceutical Technology, Johannes Gutenberg-University, Staudinger Weg 5, 55128 Mainz, Germany.

[b]European Molecular Biology Laboratory, Hamburg Unit, Notkestrasse 85, 22607 Hamburg, Germany.

[c]Postnova Analytics GmbH, Rankingstrasse 1, 86899 Landsberg am Lech, Germany.

[*]**Corrosponding Author:** Prof. Dr. Peter Langguth. E-Mail: langguth@uni-mainz.de

Table of contents

Figure S1 Supporting materials for polystyrene sample

- A Timely stacked SAXS-Scattering curves.
- B Size from different fits.
- C Stacked Guinier-fits from MALS-data.
- D Stacked Guinier-Fits from SAXS-data.

Figure S2 Supporting materials for LNP sample

- A Timely stacked SAXS-Scattering curves.
- B Size from different fits.
- C Stacked Guinier-fits from MALS-data.
- D Stacked Guinier-Fits from SAXS-data.

Figure S3 AF4-Separation method

- A Method graph for the polystyrene particles.
- B Method graph for the LNPs.

Figure S4 Parameters derived from Bragg-peak analysis

Figure S5 Dynamic light scattering from batch measurement of mRNA LNPs. Summary of Hydrodynamic diameter (z-Average) and Polydispersity Index (PDI).

Figure S6 Workflow diagram for the experiment and data treatment

Table S1 Scattering angles to scattering vector conversion

Table S2 Detailed table including time, d-spacing, correlation length, and Bragg-AUC values.

Table S3 Fit parameters from Guinier plots with respective confidence levels

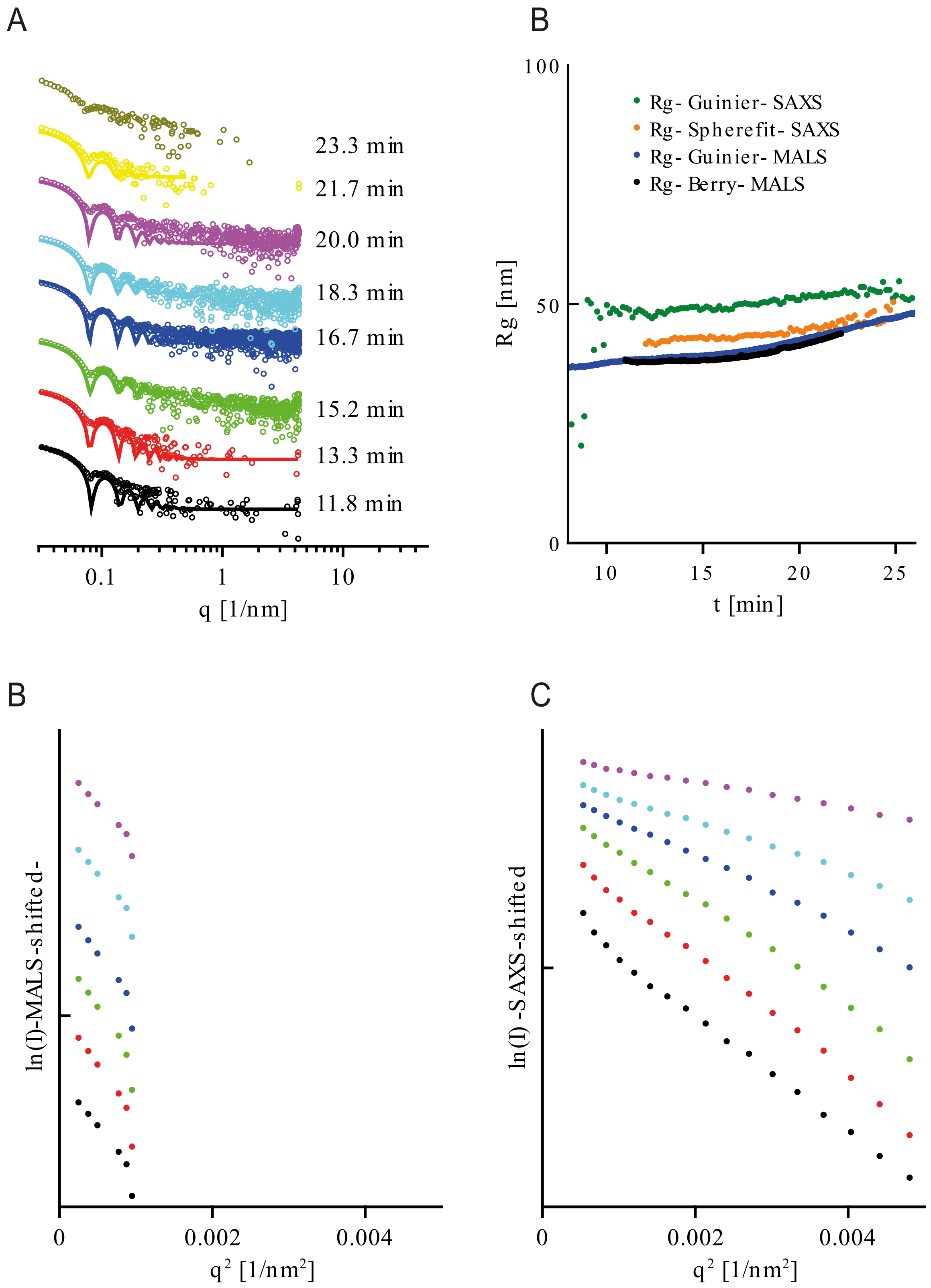

Figure S1 Supporting materials for polystyrene sample. A Timely stacked SAXS-Scattering curves. B Size from different fits. C Stacked Gunier-fits from MALS-data. D Stacked Guinier-Fits from SAXS-data.

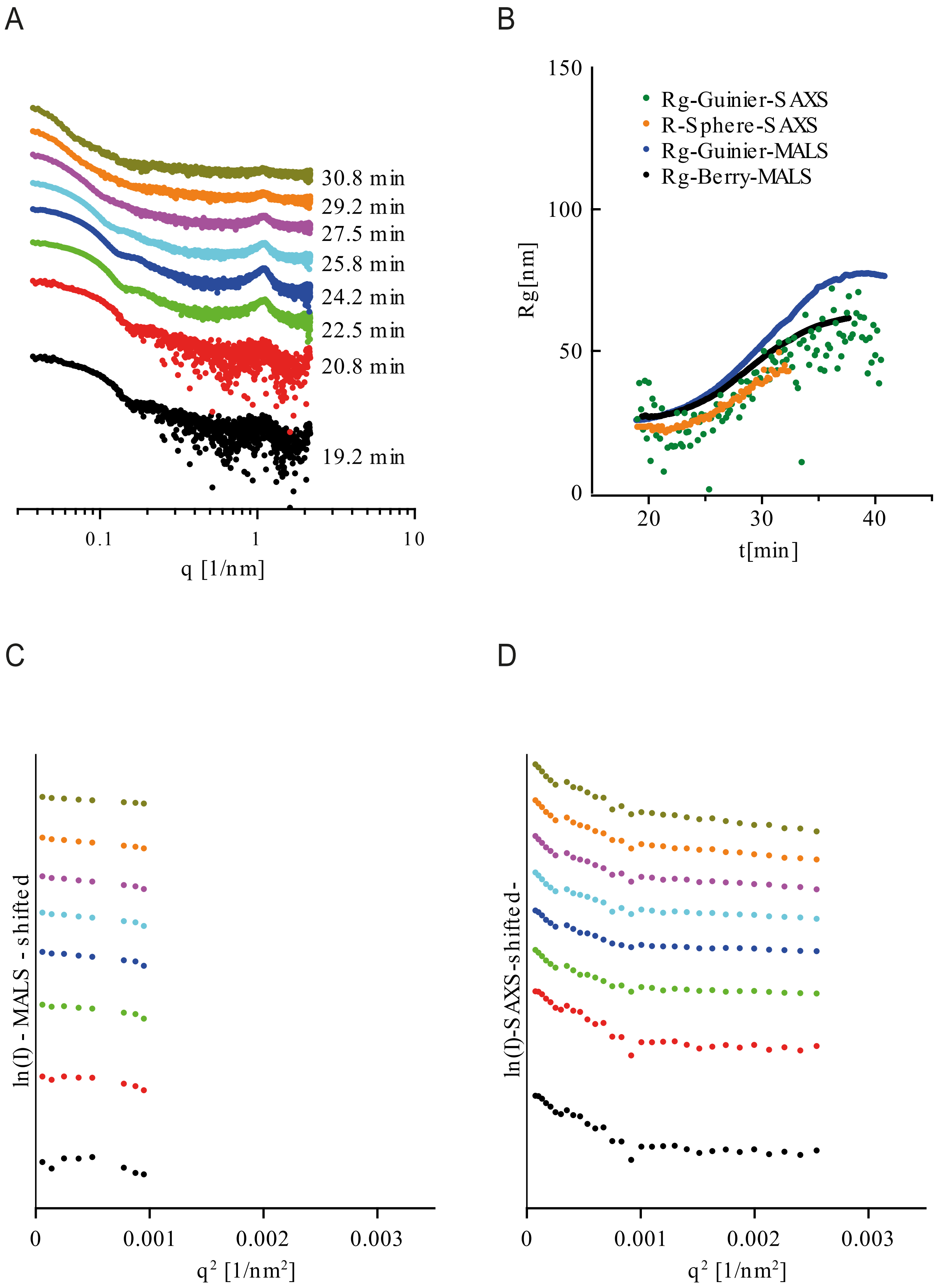

Figure S2 Supporting materials for LNP sample. A Timely stacked SAXS-Scattering curves. B Size from different fits. C Stacked Gunier-fits from MALS-data. D Stacked Guinier-Fits from SAXS-data.

A

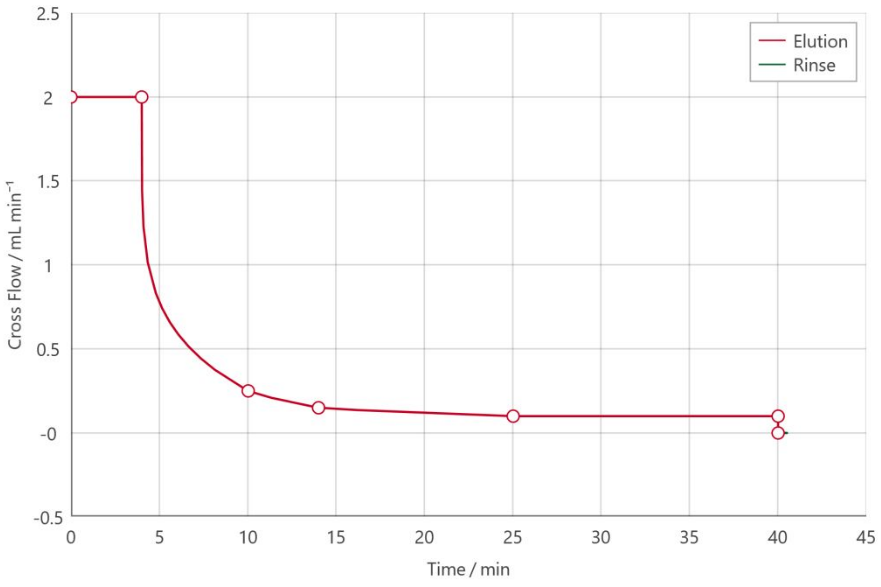

B

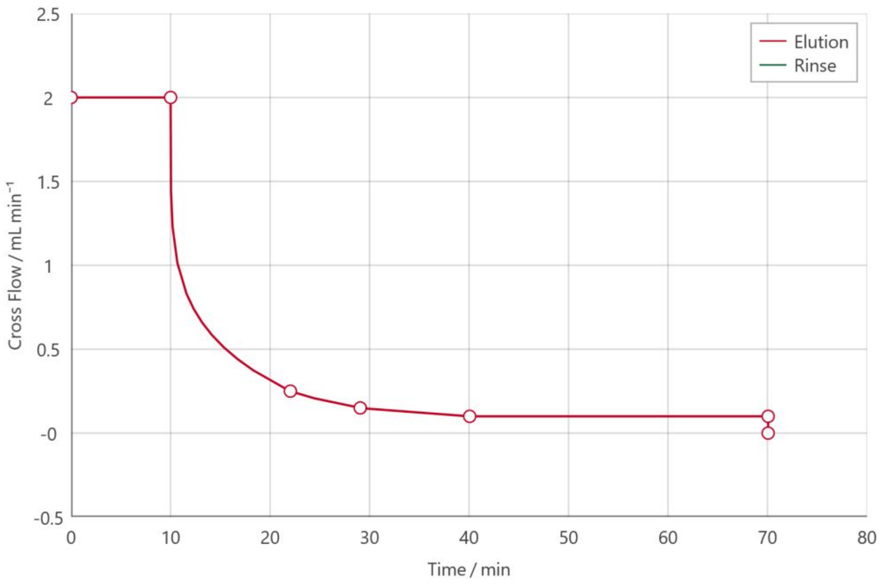

Figure S3 AF4-Separation method: Figure B displays the method graph for the LNPs and Figure A for the polystyrene particles.

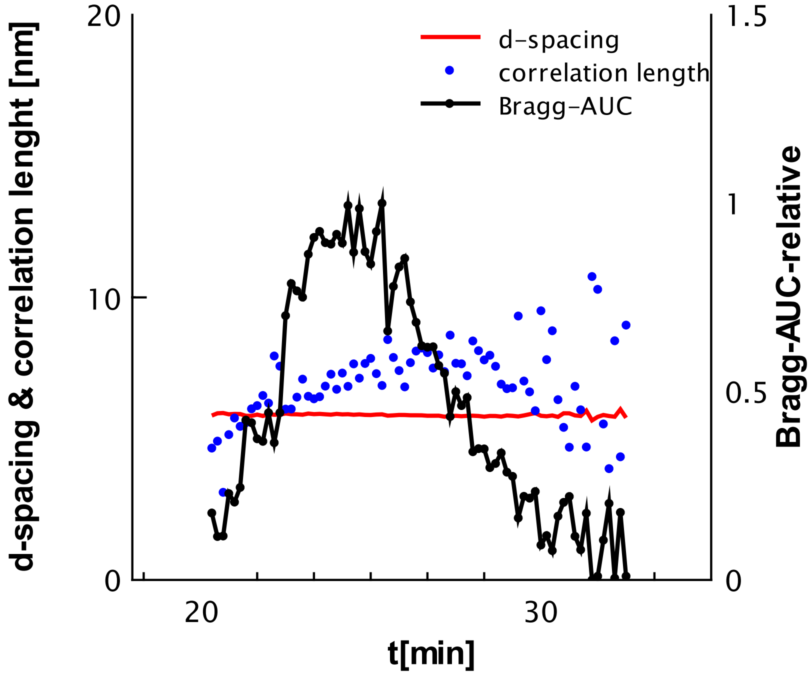

Figure S4 Display of parameters from Bragg analysis

Results

			Size (d.n...	% Intensity:	St Dev (d.n...
**Z-Average (d.nm):**	110.8	**Peak 1:**	124.2	100.0	43.40
**PdI:**	0.104	**Peak 2:**	0.000	0.0	0.000
**Intercept:**	0.964	**Peak 3:**	0.000	0.0	0.000
**Result quality**	**Good**				

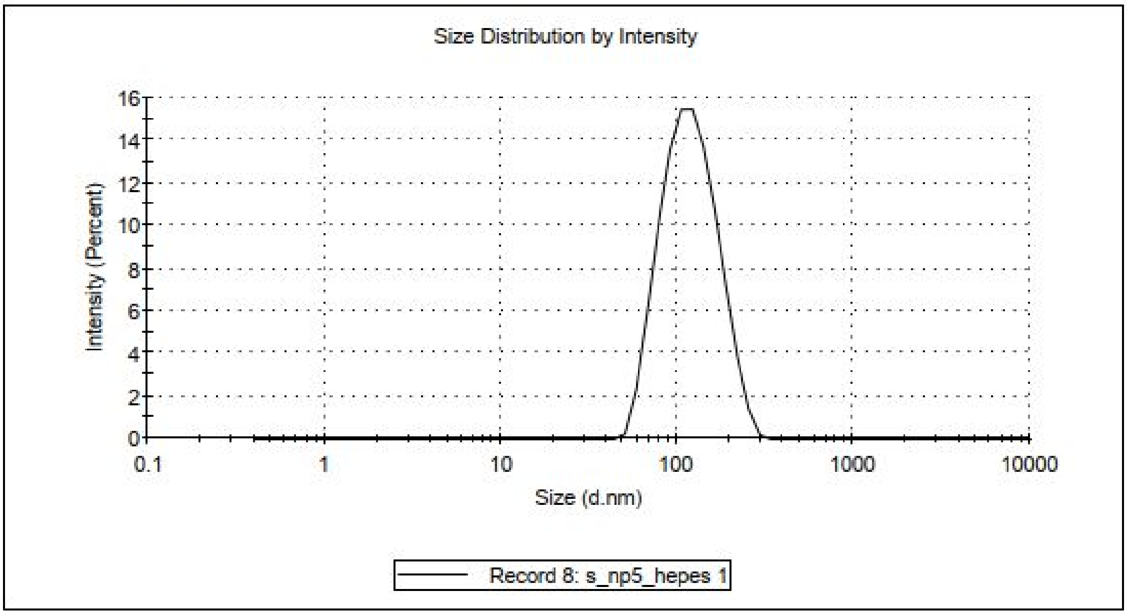

Figure S5 Dynamic light scattering data from batch measurement of mRNA LNP. Hydrodynamic diameter (z-Average) = 110.8 nm and a dispersity (PDI) = 0.104. Temperature T= 25°C ; Size runs n = 14

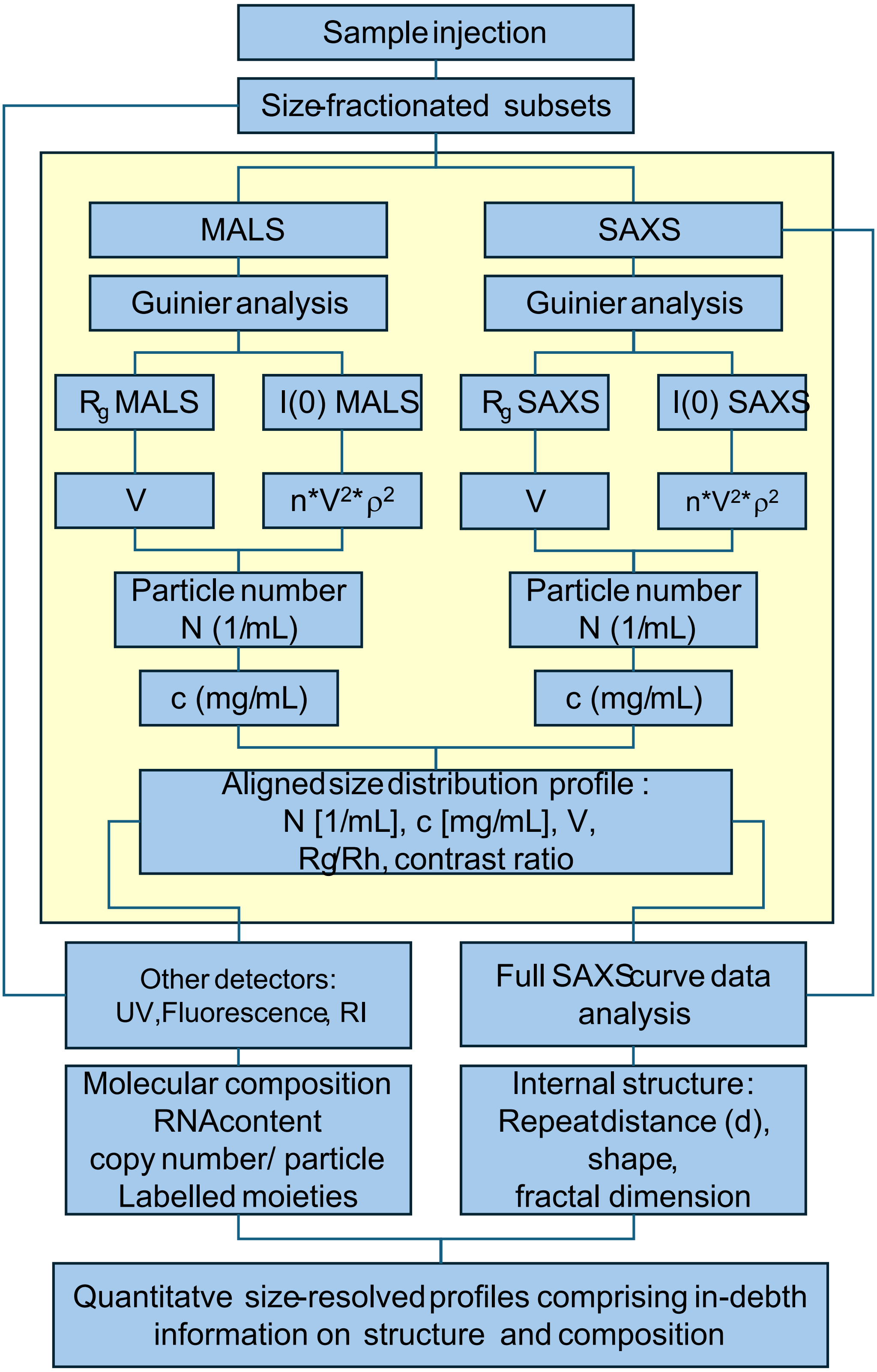

Figure S6 Workflow diagram. The yellow area highlights the harmonized data treatment done in the manuscript.

Scattering angle Θ [°]	Scattering vector q [1/nm]
28	7.62E-03
44	1.18E-02
60	1.57E-02
76	1.94E-02
90	2.23E-02
124	2.78E-02
140	2.96E-02
156	3.08E-02

Table S1 scattering angles to scattering vector conversion with λ = 532 nm in water

time [min]	d-spacing [nm]	correlation length [nm]	Bragg-AUC [nm-2]	time [min]	d-spacing [nm]	correlation length [nm]	Bragg-AUC [nm-2]
20.3	5.8	4.7	1.7	26.5	5.8	8.2	4.4
20.5	5.9	4.9	1.3	26.7	5.8	8.0	4.4
20.7	5.9	3.1	1.3	26.8	5.8	7.5	4.4
20.8	5.9	5.1	2.0	27.0	5.8	8.0	4.1
21.0	5.9	5.7	1.8	27.2	5.8	7.4	3.9
21.2	5.9	5.4	2.1	27.3	5.8	8.7	3.2
21.3	5.8	5.6	3.2	27.5	5.8	7.7	3.6
21.5	5.8	6.1	3.1	27.7	5.8	7.7	3.4
21.7	5.9	6.2	2.9	27.8	5.8	7.2	3.5
21.8	5.8	6.5	2.8	28.0	5.8	8.5	2.7
22.0	5.9	6.3	3.3	28.2	5.8	8.1	2.7
22.2	5.8	7.9	2.8	28.3	5.8	7.8	2.7
22.3	5.9	7.6	3.3	28.5	5.8	8.0	2.4
22.5	5.9	6.0	4.9	28.7	5.8	7.6	2.5
22.7	5.9	6.1	5.4	28.8	5.8	6.9	2.6
22.8	5.9	6.5	5.3	29.0	5.8	6.8	2.3
23.0	5.8	7.1	5.2	29.2	5.8	6.8	2.3
23.2	5.9	6.5	5.9	29.3	5.8	9.3	1.6
23.3	5.9	6.4	6.2	29.5	5.8	7.0	1.9
23.5	5.9	6.5	6.3	29.7	5.9	6.7	1.9
23.7	5.9	6.9	6.1	29.8	5.9	6.0	2.0
23.8	5.9	7.3	6.1	30.0	5.8	9.5	1.1
24.0	5.9	6.7	6.2	30.2	5.8	7.8	1.3
24.2	5.9	7.3	6.1	30.3	5.8	8.8	1.0
24.3	5.9	6.9	6.7	30.5	5.8	6.4	1.6
24.5	5.8	7.6	5.9	30.7	5.9	5.4	1.8
24.7	5.9	7.1	6.6	30.8	5.9	4.7	1.9
24.8	5.8	7.7	5.9	31.0	5.8	6.9	1.3
25.0	5.8	7.8	5.7	31.2	5.8	6.0	1.1
25.2	5.9	7.3	6.3	31.3	6.0	4.7	1.7
25.3	5.9	6.9	6.7	31.5	5.6	10.7	0.6
25.5	5.8	8.5	4.6	31.7	5.8	10.3	0.6
25.7	5.8	7.9	5.4	31.8	5.8	5.5	1.2
25.8	5.8	7.4	5.7	32.0	5.8	3.9	1.8
26.0	5.8	6.8	5.8	32.2	5.8	8.5	0.6
26.2	5.8	7.7	5.1	32.3	6.0	4.4	1.7
26.3	5.8	8.1	4.8	32.5	5.7	9.0	0.6

Table S2 Parameters derived from Bragg-peak analysis

Sample - Dataset - Time	Fit parameter	Value	95% LCL	95% UCL
Polystyrol -SAXS	slope	-7.73E+02	-8.00E+02	-7.47E+02
	intercept	-1.27E+00	-1.33E+00	-1.22E+00
Polystyrol -MALS	slope	-5.15E+02	-5.51E+02	-4.80E+02
	intercept	-4.24E+00	-4.26E+00	-4.23E+00
LNP-SAXS - 24.5min	slope	-2.48E+02	-3.43E+02	-1.53E+02
	intercept	-3.29E+00	7.25E-02	-1.53E+02
LNP-MALS - 24.5min	slope	-3.64E+02	-4.10E+02	-3.19E+02
	intercept	-6.05E+00	-6.08E+00	-6.03E+00
LNP-SAXS - 35.0min	slope	-1.15E+03	-1.38E+03	-9.30E+02
	intercept	-2.88E+00	-3.24E+00	-2.53E+00
LNP-MALS - 35.0min	slope	-1.76E+03	1.62E+02	-1.31E+03
	intercept	-6.35E+00	-6.60E+00	-6.10E+00

Table S3 Fit parameters from Guinier plots with respective confidence levels (confidence interval = 95%)
